# Delineating visual, auditory and motor regions in the human brain with functional neuroimaging: a BrainMap-based meta-analytic synthesis

**DOI:** 10.1038/s41598-021-88773-9

**Published:** 2021-05-11

**Authors:** Marisa K. Heckner, Edna C. Cieslik, Vincent Küppers, Peter T. Fox, Simon B. Eickhoff, Robert Langner

**Affiliations:** 1 Institute of Neuroscience and Medicine (INM-7: Brain and Behaviour), Research Centre Jülich, 52425 Jülich, Germany; 2 Institute of Systems Neuroscience, Medical Faculty, Heinrich Heine University Düsseldorf, Düsseldorf, Germany; 3 Research Imaging Center, University of Texas Health Science Center, San Antonio, TX USA

**Keywords:** Cognitive neuroscience, Neuroscience, Sensory processing

## Abstract

Most everyday behaviors and laboratory tasks rely on visual, auditory and/or motor-related processes. Yet, to date, there has been no large-scale quantitative synthesis of functional neuroimaging studies mapping the brain regions consistently recruited during such perceptuo-motor processing. We therefore performed three coordinate-based meta-analyses, sampling the results of neuroimaging experiments on visual (n = 114), auditory (n = 122), or motor-related (n = 251) processing, respectively, from the BrainMap database. Our analyses yielded both regions known to be recruited for basic perceptual or motor processes and additional regions in posterior frontal cortex. Comparing our results with data-driven network definitions based on resting-state functional connectivity revealed good overlap in expected regions but also showed that perceptual and motor task-related activations consistently involve additional frontal, cerebellar, and subcortical areas associated with “higher-order” cognitive functions, extending beyond what is captured when the brain is at “rest.” Our resulting sets of domain-typical brain regions can be used by the neuroimaging community as robust functional definitions or masks of regions of interest when investigating brain correlates of perceptual or motor processes and their interplay with other mental functions such as cognitive control or affective processing. The maps are made publicly available via the ANIMA database.

## Introduction

Visual, auditory, and motor-related processes are important for most everyday behaviors as well as laboratory tasks. In fact, “basic” perceptual and motor processing may play a substantive role in “higher-order” mental faculties such as cognitive control. Research on cognitive aging has already implicated a significant link between perceptual and cognitive functions. For example, the processing-speed theory of adult age differences in cognition^1^ postulates that slowing of “higher-order” cognitive functions in older adults might be caused by poorer utilization of cognitive resources at an earlier processing level. Poorer performance in “higher-order” cognitive tasks might thus be a consequence of inefficient sensory and/or motor processing^2,3^. However, especially neuroimaging studies that focus on network-based substrates of cognitive functions often do not account for perceptuo-motor processes and their neural correlates, thereby ignoring regions whose processing might be crucial but not specific for the process of interest. One reason for this might be that a great number of networks of interest result from task-based neuroimaging studies, which typically follow a subtraction logic that “cancels out” any brain activation that is not specifically affected by the condition of interest, relative to a control condition, but may nonetheless be essential for solving the task at hand. This is particularly true of perceptuo-motor processes, which often precede, follow or accompany the mental process under scrutiny and are crucial for correct task performance^4,5^. For revealing their neural mechanisms with ecological validity, it seems useful to jointly address context-, input-, and output-related subprocesses, as opposed to focusing on single, isolated core functions. Such an integration of perceptual and motor-related processes in research on higher cognitive functions in the normal population still seems to be largely missing, though.

To date, there has not been any large-scale quantitative synthesis of functional neuroimaging studies mapping the brain regions recruited during such perceptuo-motor processing. Previous meta-analyses^6,7^ might suffer from a lack of power and generalizability, as they included less than half the number of experiments used in the current study and focused on rather specific subprocesses within their functional domains. This study therefore aimed to robustly and broadly define perceptual and motor-related brain regions as well as a general perceptuo-motor network. To do so, we used activation likelihood estimation (ALE) meta-analyses^8–11^ for synthesizing results from neuroimaging studies investigating visual or auditory processing as well as motor execution. While we focused on rather simple and “pure” perceptual or motor paradigms, to capture regions associated with fundamental perceptuo-motor processes, we did not limit our analyses to specific subprocesses or functions. Rather, our experiment selection aimed to be as comprehensive as possible to distill what is shared across a great number and variety of domain-specific tasks to allow for a robust and unbiased estimation.

Imaging data was obtained from the BrainMap database (http://www.brainmap.org)^12–15^, which stores peak coordinates from published neuroimaging studies along with metadata describing the paper. In order to capture brain activations associated with perceptuo-motor processes, our meta-analyses only included experiments that did not cancel out these basic processes. That is, we excluded all experiments that performed subtractions with sensory or motor control conditions, respectively. The term “experiment” refers to a single contrast between imaging data yielding condition-specific localization information. A scientific publication can report one or more experiments^15^. Our definition of perceptuo-motor processes was based on BrainMap’s taxonomy of *Paradigm Classes*, that is, a set of descriptive labels categorizing the experimental tasks^16^. For example, for visual processes we included, among other paradigms: *film viewing*, *fixation*, and *flashing checkerboard*; for auditory processes we included paradigms such as *passive listening*, *music comprehension* and *pitch monitor*; and for motor execution we included paradigms such as *chewing*, *grasping*, and *finger tapping* (see Methods for a full list).

In a first step, the neural correlates of visual, auditory or motor-related processes were examined in three separate ALE meta-analyses. In a second step, the maximum z-statistic of the three resulting sets of regions was computed to obtain a general perceptuo-motor network. In a third step, we compared our meta-analytically derived task-based networks to previously published definitions of brain networks based on data-driven analyses of resting-state functional connectivity (RSFC). Since it has been shown that task-related brain activation patterns are mirrored by functional connectivity patterns at rest^17–20^, the comparison to previously published resting-state networks was used as a means to assess the validity of our task-based network definitions. Further, this comparison enabled us to distinguish brain regions associated with “basic” perceptuo-motor processes (i.e., brain regions that are common between our task-based and at least one RS-derived network) from domain-unspecific regions linked to task execution and/or setting. Finally, we computed a minimum z-statistic conjunction across visual, auditory and motor networks to identify regions that are domain-general (i.e., independent of fundamental perceptuo-motor processes).

## Methods

### Sample

Using Sleuth 3.0.3 (http://www.brainmap.org/sleuth/), we identified relevant functional imaging experiments in the BrainMap database^12–15^, which contained 16,901 experiments at the time of analysis. We only considered activation data from functional magnetic resonance imaging (fMRI) or positron emission tomography (PET) studies with healthy adult participants. Furthermore, we only included experiments with whole-brain coverage (i.e., no results of region-of-interest analyses) and low-level control condition (i.e., task > resting baseline). We excluded results of correlation analyses with external variables and between-group contrasts. To be precise, our exclusion criteria were applied to experimental conditions and not the paper per se. Hence, if a paper reported a whole-brain analysis that met the criteria but also reported (additional) post-hoc region-of-interest analyses, the whole-brain results were still included.

**Figure 1:**
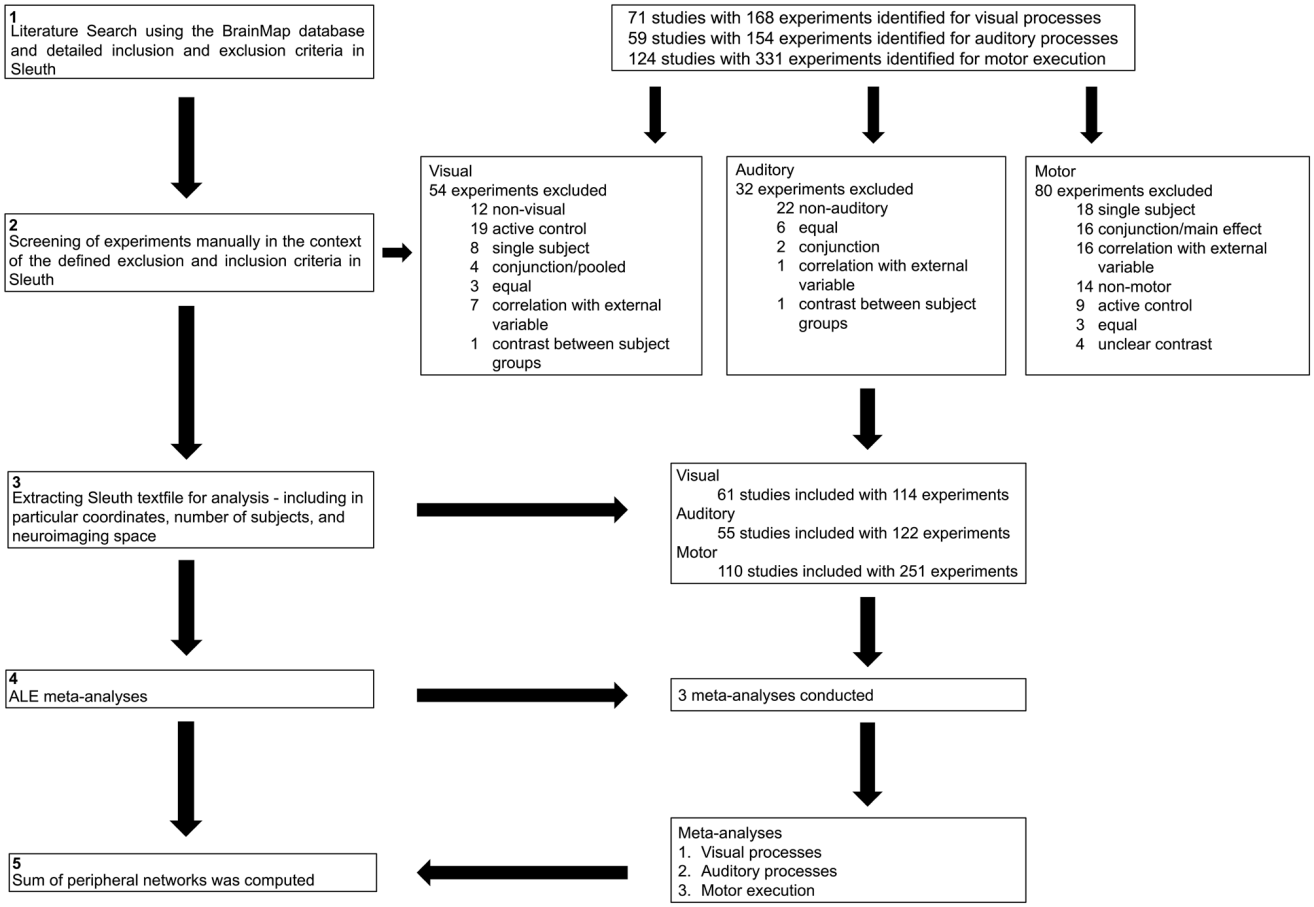
Flowchart of the meta-analysis steps conducted.

In BrainMap, experimental tasks are coded along two dimensions^16^: *Behavioral Domains* describe the cognitive process probed by an experiment, and *Paradigm Classes* label the task category used. Here, we selected all *Paradigm Classes* that captured basic sensory visual or auditory input processing or relatively simple motor execution processes, respectively. After an initial automated extraction, experiments were further screened in BrainMap’s *Workspace* by the first author to double-check their match with our inclusion and exclusion criteria. In particular, it was ascertained that the visual, auditory and motor-related processes of our interest were not subtracted out by an active sensory or motor baseline condition (see Fig. 1 for an overview of the different analysis steps conducted). A checklist for neuroimaging meta-analyses^21^ including detailed information about the automated and manual inclusion and exclusion criteria can be found in Supplementary Table S1.

For elucidating regions consistently involved in visual processing, we included experiments of the following *Paradigm Classes: face monitor/discrimination*, *film viewing*, *fixation*, *flashing checkerboard*, *passive viewing*, *visual object identification*, *visual pursuit/tracking*, and *visuospatial attention*. The automated extraction resulted in 168 experiments, 114 of which were included in our analysis after further manual screening.

For identifying regions consistently involved in auditory processing, we included experiments of the *Paradigm Classes: divided auditory attention*, *music comprehension*, *oddball discrimination*, *passive listening*, *phonological discrimination*, *pitch monitor/discrimination* and *tone monitor/discrimination*. The automated extraction resulted in 154 experiments, 122 of which were included in our analysis after further manual screening.

For determining regions consistently involved in motor-related processing, we included experiments of the *Paradigm Classes: writing*, *chewing/swallowing*, *drawing, isometric force*, *motor learning*, *grasping*, *finger tapping/button press*, and *flexion/extension.* The automated extraction resulted in 331 experiments, 251 of which were included in our analysis after further manual screening.

### Activation likelihood estimation

The meta-analyses were conducted using the revised version of the ALE algorithm for coordinate-based meta-analysis of neuroimaging results as implemented in GingerALE 3.0.2^8,9,11^. This algorithm aims to identify areas with across-experiment activity convergence that is higher than expected from random spatial association. ALE models the activation coordinates included as centers of 3-D Gaussian probability distributions to acknowledge the spatial uncertainty associated with each focus, which is weighted according to the number of participants per experiment. The probability distributions of all activation foci of a given experiment are then combined for each voxel, which creates a modeled activation (MA) map. Taking the union across these MA maps of all experiments included yields voxel-wise ALE scores describing the convergence of results (i.e., the estimated activation likelihood) across studies at each particular location of the brain. These ALE scores are then compared to an empirical null distribution reflecting random spatial associations between all MA maps to distinguish “true” convergence across studies from random convergence. A detailed description can be found in the Supplementary Material. All results were thresholded at *p* < 0.05 (cluster inclusion threshold at voxel level: *p* < 0.001).

In addition to the three individual meta-analyses, we combined their results by calculating a voxel-wise maximum z-statistic on the thresholded result images (i.e., the greatest z-value per voxel of each of the three images was retained) to define a comprehensive perceptuo-motor processing network. Furthermore, we computed a voxel-wise minimum z-statistic on the thresholded result images to display common regions between all three perceptuo-motor domains. All results were anatomically labeled by reference to probabilistic cytoarchitectonic maps of the human brain using the SPM Anatomy Toolbox version 3^22,23^. Cortical and cerebellar clusters of convergence were visualized with the BrainNet Viewer^24^; subcortical clusters were rendered on the individual anatomical template (“ch2better”) provided with MRIcron^25^.

### Comparison to RSFC-based data-driven network definitions

For comparing our meta-analytically derived ALE maps to RSFC-based data-driven network definitions we used the Jaccard similarity coefficient^26,27^. The Jaccard coefficient as a comparison of two binarized activation maps is the intersection divided by the union of all voxels [Jaccard = C/(A + B − C), where C is the number of significant voxels at the intersection of both maps and A and B the number of significant voxels of each map]. A Jaccard coefficient of 1 indicates perfect between-map overlap, a coefficient of 0 no overlap.

In particular, we compared our results with Yeo et al.’s^28^ networks reflecting an RSFC-based 7-cluster parcellation of the cortex, with Smith et al.’s^17^ networks reflecting a 20-component decomposition obtained with independent component analysis, and Power et al.’s^29^ subnetworks obtained with a subgraph detection algorithm.

As there was no 3D-image of the Power networks available, we calculated the overlap as number of Power network coordinates within the corresponding ALE-derived network divided by the total number of nodes comprising the respective Power network. For visualization of the overlap, task-based and the corresponding resting-state derived brain networks were rendered on the individual anatomical template (“ch2better”) provided with MRIcron^25^. Venn diagrams were created using the Python package Matplotlib-venn.

## Results

### Meta-analyses

**Figure 2:**
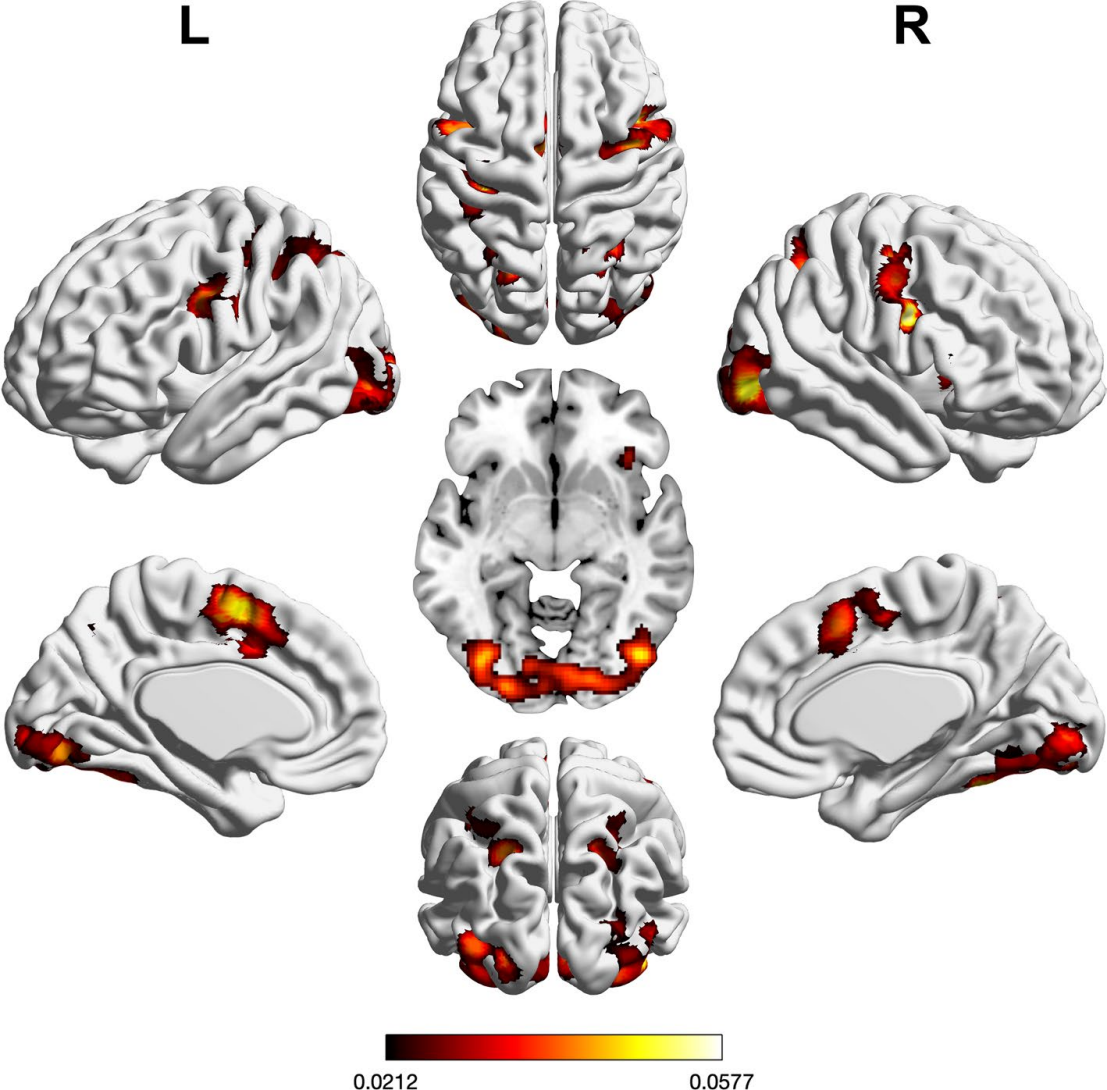
Foci of brain activity showing significant across-experiment convergence of activity related to visual processing (cluster-level *p* < 0.05, family-wise error-corrected for multiple comparisons, cluster-forming threshold at voxel level: *p* < 0.001). The scale bar reflects activation likelihood estimation scores. Cortical and cerebellar clusters of convergence were visualized with the BrainNet Viewer^24^; subcortical clusters were rendered on the individual anatomical template (“ch2better”) provided with MRIcron^25^.

We performed three ALE meta-analyses to uncover the neural correlates of visual or auditory input processing as well as motor execution. Regions of significant convergence across visual tasks are shown in Fig. 2 and Supplementary Table S2. In brief, convergence was found in bilateral visual cortices, fusiform gyrus, pre-supplementary motor area (preSMA), inferior frontal junction (IFJ), intraparietal sulcus (IPS), superior parietal lobule (SPL), and dorsal premotor cortex (PMd) including the right frontal eye field (FEF). Further convergence was observed in left lingual gyrus and left SMA as well as right anterior insula (aINS).

**Figure 3:**
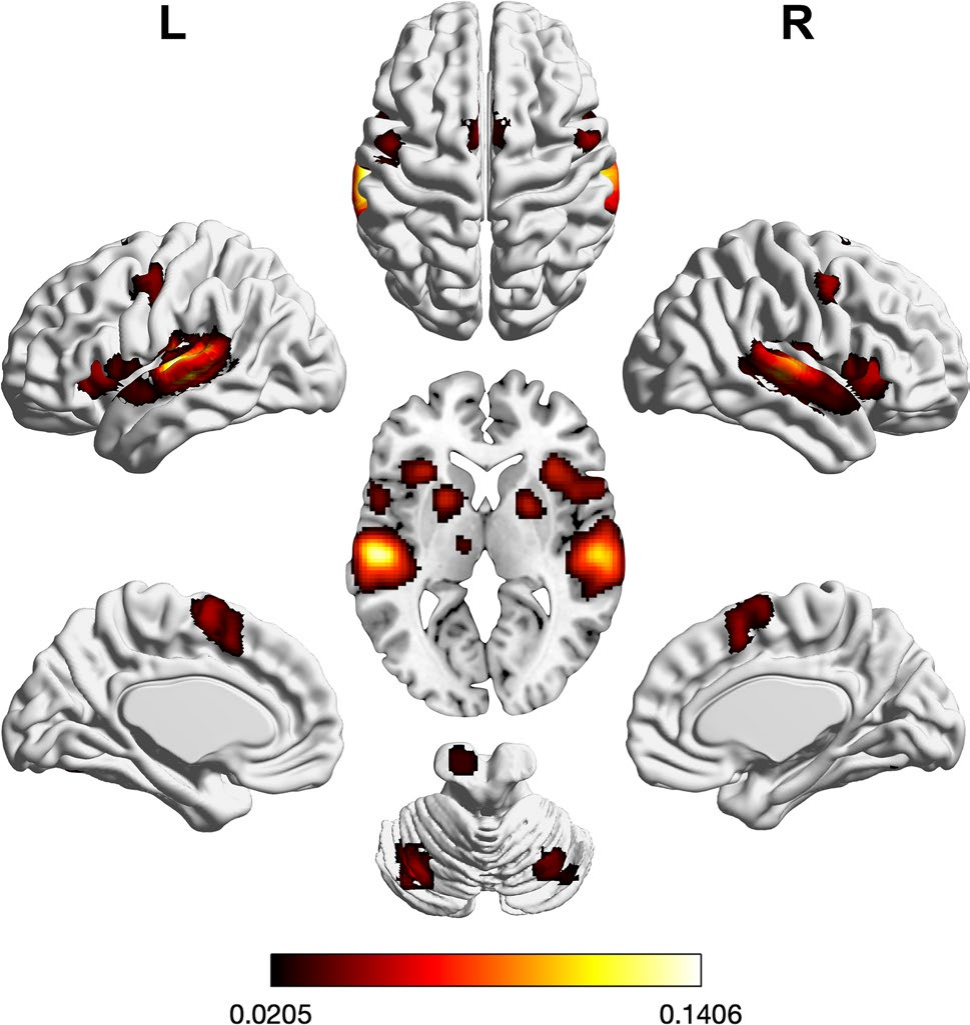
Foci of brain activity showing significant convergence of activity for auditory processes (cluster-level *p* < 0.05, family-wise error-corrected for multiple comparisons, cluster-forming threshold at voxel level: *p* < 0.001). The scale bar reflects activation likelihood estimation scores. Cortical and cerebellar clusters of convergence were visualized with the BrainNet Viewer^24^; subcortical clusters were rendered on the individual anatomical template (“ch2better”) provided with MRIcron^25^.

Regions of significant convergence across tasks taxing auditory processing were found in bilateral planum temporale, Heschl’s gyrus, aINS, superior temporal gyrus, pre-SMA, SMA, PMd/FEF, and inferior frontal gyrus (IFG) pars opercularis, as well as putamen, thalamus, and cerebellum (see Supplementary Table S3 and Fig. 3).

**Figure 4:**
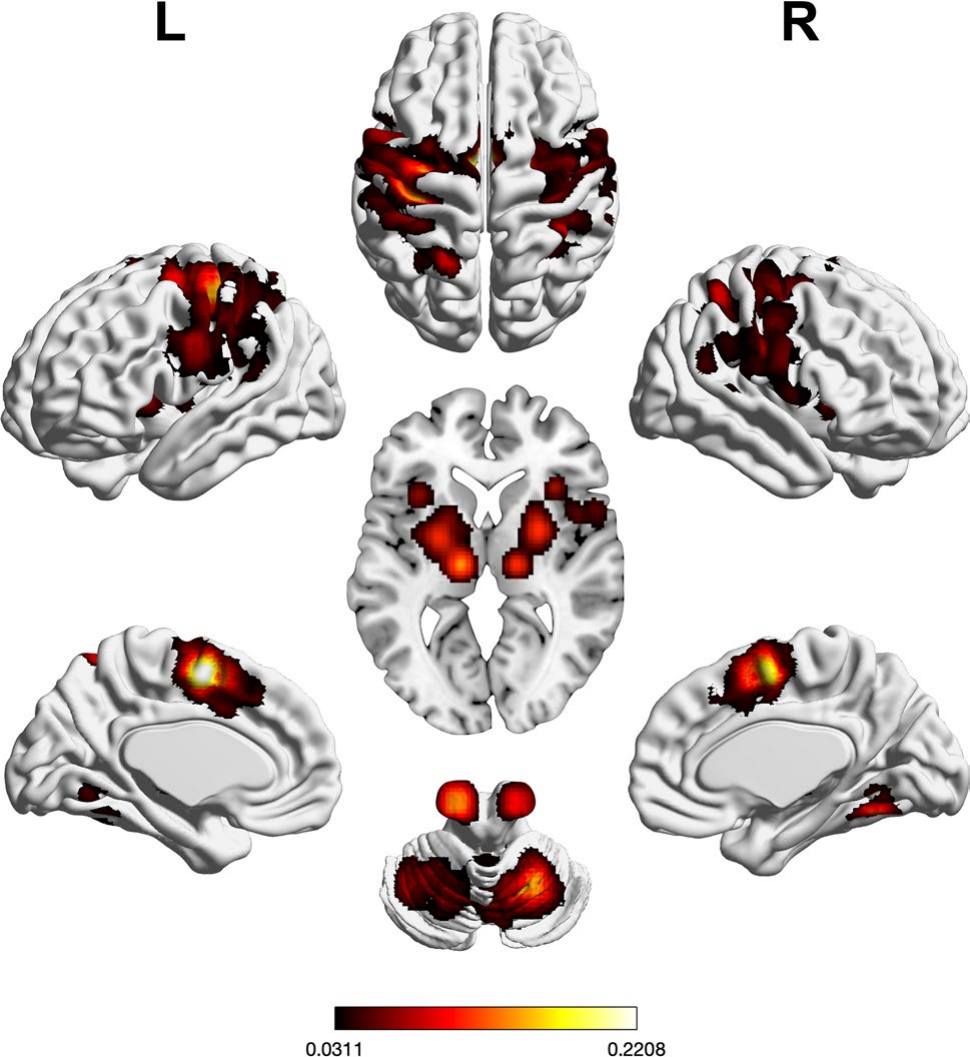
Foci of brain activity showing significant convergence of activity for motor execution (cluster-level *p* < 0.05, family-wise error-corrected for multiple comparisons, cluster-forming threshold at voxel level: *p* < 0.001). The scale bar reflects activation likelihood estimation scores. Cortical and cerebellar clusters of convergence were visualized with the BrainNet Viewer^24^; subcortical clusters were rendered on the individual anatomical template (“ch2better”) provided with MRIcron^25^.

Regions of significant convergence across tasks probing motor processes were found in bilateral pre-SMA, SMA, PMd including FEF, primary motor and somatosensory cortex, IPS, SPL, posterior IFG, and aINS, as well as putamen, thalamus, and cerebellum. Further convergence was observed in left lingual gyrus and left pallidum (see Supplementary Table S4 and Fig. 4).

**Figure 5:**
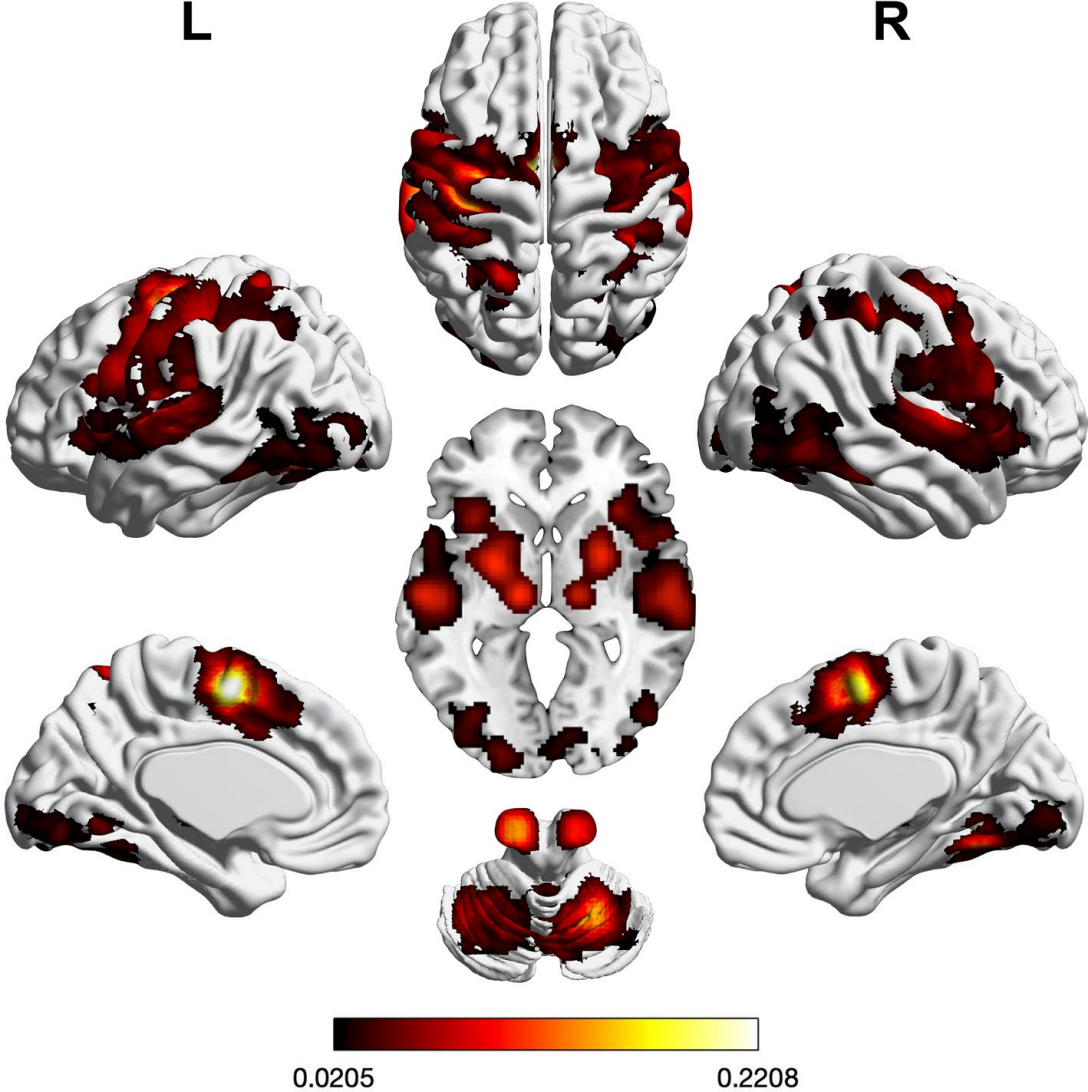
Union (maximum z-statistic) of the results of three meta-analyses on visual, auditory, and motor-related processing. The scale bar reflects the maximum statistic of the activation likelihood estimation scores. Cortical and cerebellar clusters of convergence were visualized with the BrainNet Viewer^24^; subcortical clusters were rendered on the individual anatomical template (“ch2better”) provided with MRIcron^25^.

Taking the maximum z-statistic of the resulting three sets of regions related to visual, auditory, or motor processing, respectively, yielded a comprehensive perceptuo-motor network. This combined set of regions is depicted in Fig. 5 and Supplementary Table S5.

The minimum z-statistic on the thresholded result images revealed preSMA, right aINS, and bilateral PMd as regions commonly involved in visual, auditory, as well as motor processing (see Supplementary Table S6 and Fig. S1).

### Comparison to RSFC-based data-driven network definitions

**Figure 6:**
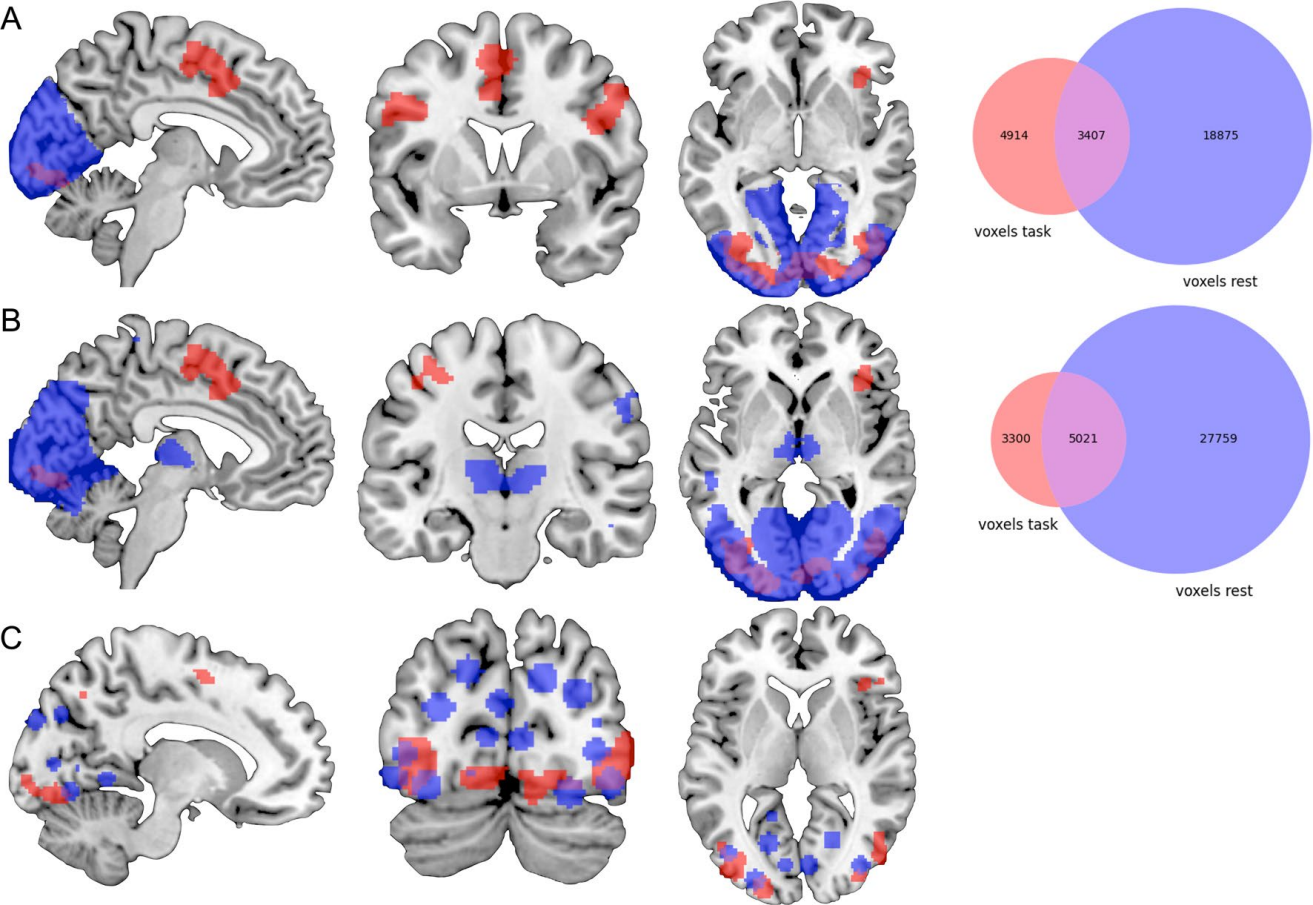
Overlap between the task-based (red) and resting-state-derived (blue) visual networks by (**A**) Yeo et al.^28^, (**B**) Smith et al.^17^, and (**C**) Power et al.^29^. For Power et al.’s graph nodes spheres of 6 mm were added for illustrative purposes. The Venn diagrams on the right illustrate the number of voxels either network as well as the overlap in between these networks comprises. Brain networks were rendered on the individual anatomical template (“ch2better”) provided with MRIcron^25^. Venn diagrams were created using the Python package Matplotlib-venn.

We compared our meta-analytically derived task-activation networks to results obtained with three distinct data-driven approaches to defining brain networks based on interregional RSFC using the Jaccard similarity coefficient. Our visual activation network (8,321 voxels) overlapped with Yeo et al.’s RSFC-based network (22,282 voxels) in visual cortices and right IPS. However, their network was more extensive in visual cortex, while our results additionally included regions of convergence in preSMA, bilateral IFJ, left IPS, and right FEF. The Jaccard similarity coefficient was 0.13 (see Fig. 6). Similarly, our motor-related activation network (19,513 voxels) also overlapped well with Yeo et al.’s RSFC-based somatomotor network (19,476 voxels), which again was more extensive throughout somatosensory cortex proper. Our results, in turn, included additional regions of convergence in bilateral putamen, thalamus, and cerebellum, which Yeo et al.’s clustering approach of the cerebral cortex could not account for. The Jaccard similarity coefficient was 0.15 (see Fig. 8). Furthermore, their 7-cluster parcellation did not yield a separate auditory network.

**Figure 7:**
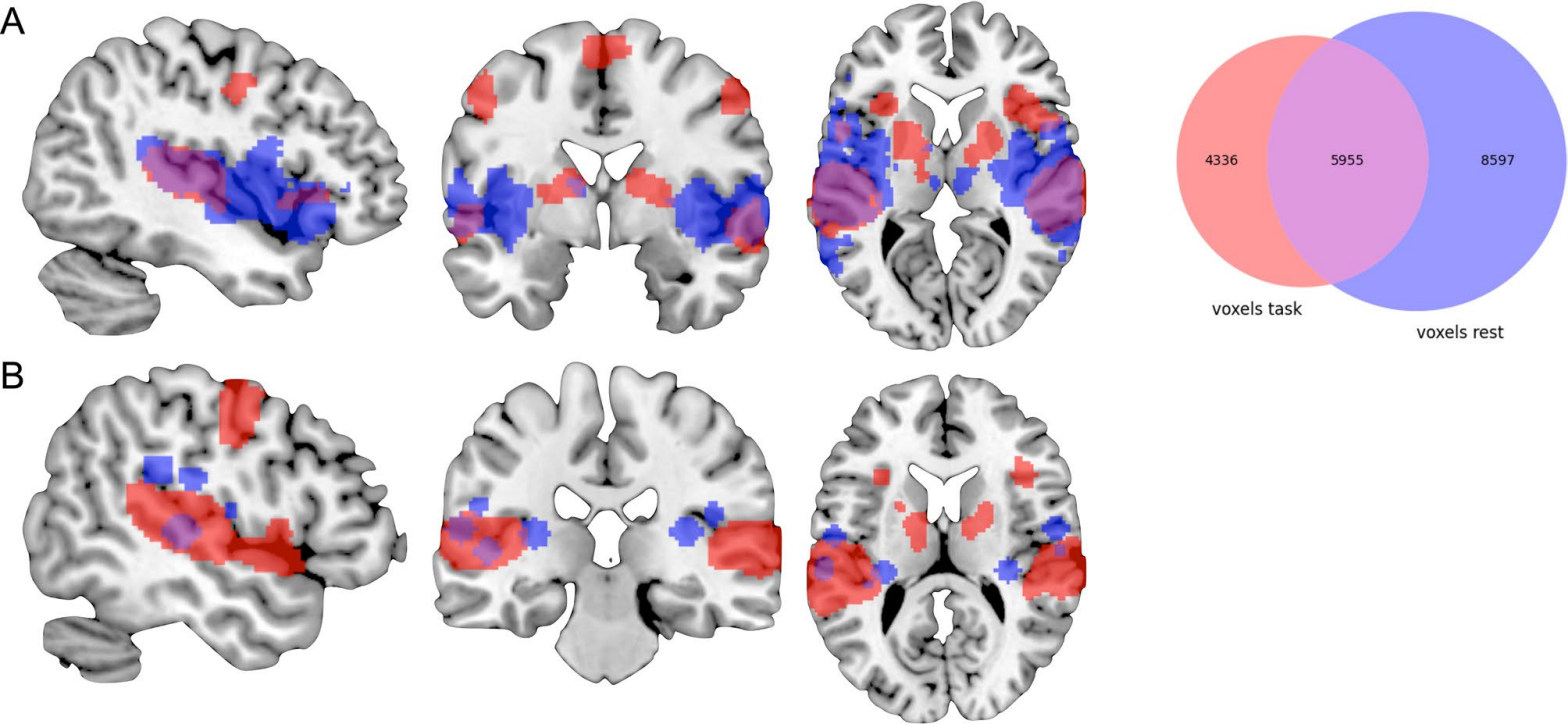
Overlap between the task-based (red) and resting-state-derived (blue) auditory networks by (**A**) Smith et al.^17^ and (**B**) Power et al.^29^. For Power et al.’s graph nodes spheres of 6 mm were added for illustrative purposes. The Venn diagram on the right illustrates the number of voxels either network as well as the overlap in between these networks comprises. Brain networks were rendered on the individual anatomical template (“ch2better”) provided with MRIcron^25^. The Venn diagram was created using the Python package Matplotlib-venn.

**Figure 8:**
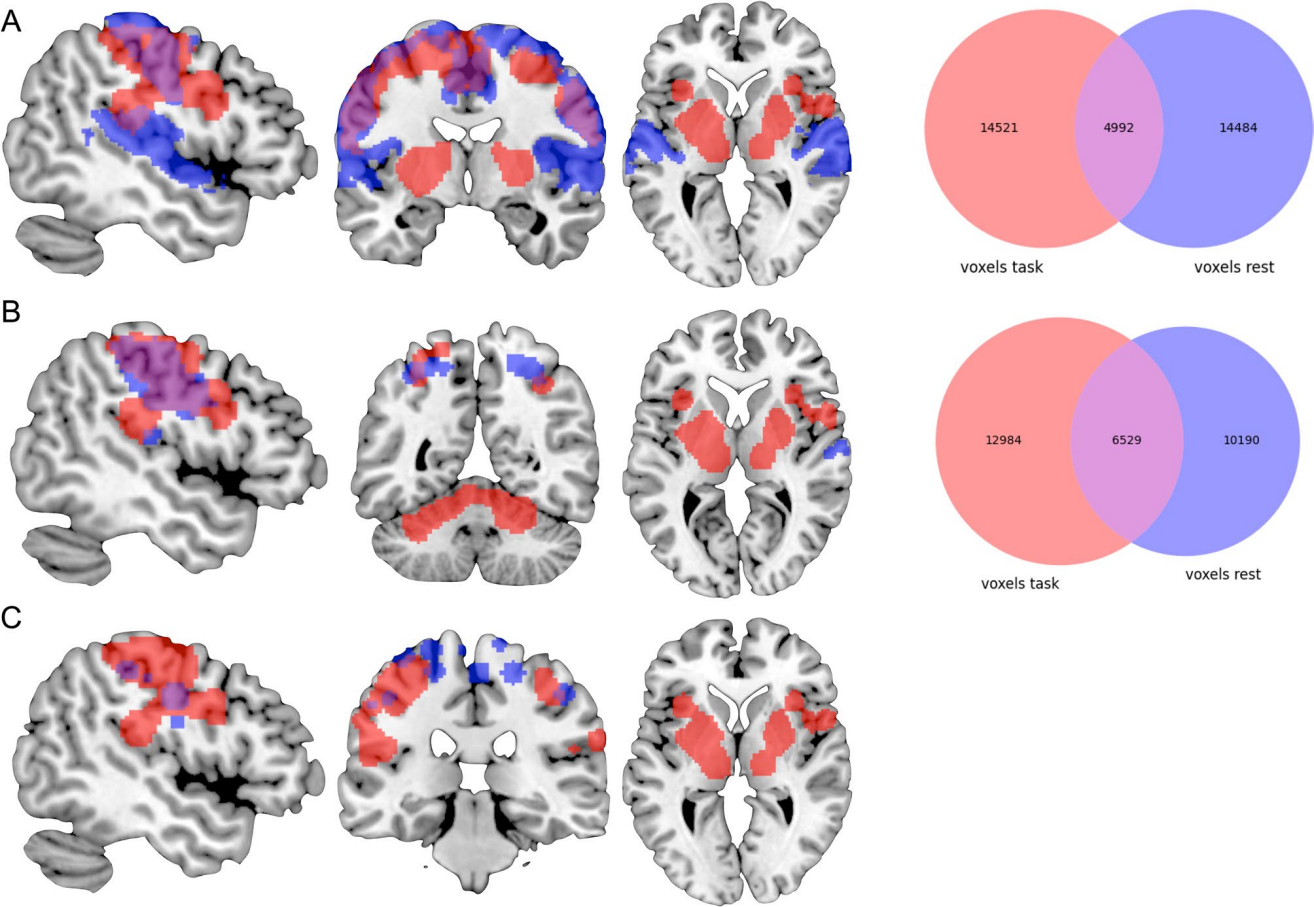
Overlap between the task-based (red) and resting-state-derived (blue) motor-related networks by (**A**) Yeo et al.^28^, (**B**) Smith et al.^17^, and (**C**) Power et al.^29^. For Power et al.’s graph nodes spheres of 6 mm were added for illustrative purposes. The Venn diagrams on the right illustrate the number of voxels either network as well as the overlap in between these networks comprises. Brain networks were rendered on the individual anatomical template (“ch2better”) provided with MRIcron^25^. Venn diagrams were created using the Python package Matplotlib-venn.

In comparison to Smith et al.’s^17^ three RSFC-derived visual networks (component nos. 1–3; 32,780 voxels), our visual-processing-related set of regions (8,321 voxels) included additional clusters of convergence in preSMA, bilateral IFJ, right IPS and right aIns, as well as in left precentral gyrus, while especially Smith et al.’s second visual network (component no. 2) included additional clusters in bilateral thalamus and was more extensive throughout pre- and postcentral gyrus. The Jaccard similarity coefficient was 0.14 (see Fig. 6). Our auditory-processing-related set of regions (10,291 voxels) comprised additional regions of convergence in bilateral cerebellum, aIns, preSMA, precentral gyrus, and right putamen. Smith et al.’s corresponding RSFC-based network (component no. 7; 14,552 voxels) included more extensive parts of primary auditory cortex. The Jaccard similarity coefficient was 0.32 (see Fig. 7). Additionally, we compared our motor-related task activation network (19,513 voxels) to Smith et al.’s somatosensory network (component no. 6; 16,719 voxels). Again, both sets of regions showed large overlap, while our results included additional regions of convergence in bilateral putamen, thalamus, and cerebellum; however, Smith et al. could not include inferior parts of the cerebellum. Conversely, Smith et al. reported an additional cluster in posterior cingulate cortex. The Jaccard similarity coefficient was 0.22 (see Fig. 8).

Finally, our task-based results also showed high overlap with Power et al.’s^29^ RSFC-based data-driven subgraphs in primary visual-, auditory-, and motor-processing-related regions. While Power et al.’s visual subgraph was more extensive throughout occipital cortex, our task-based network included additional clusters in posterior frontal regions. 6 of their 31 coordinates showed overlap with our task-based map (19%; see Fig. 6). Likewise, our auditory-processing-related set of regions comprised further posterior frontal and subcortical regions. Here, 4 of their 13 coordinates showed overlap with our network (31%; see Fig. 7). Lastly, we compared our motor-related task activation network to Power et al.’s sensory/somatomotor hand and mouth networks. The RSFC-based networks were more extensive throughout primary motor and somatosensory cortex as well as bilateral PMd and included additional regions in bilateral anterior cingulate cortex and right posterior insula. Our task-based network, on the other hand, comprised additional regions of convergence in bilateral fronto-parietal, subcortical, and cerebellar regions. 9 of their 35 coordinates showed overlap with our network (26%; see Fig. 8).

## Discussion

In this study, we conducted three large-scale coordinate-based meta-analyses to quantitatively synthesize results from neuroimaging studies on basic visual or auditory processing as well as motor execution. In a first step, we meta-analyzed the neural correlates of these three different processes separately. In a second step, we computed the maximum z-statistic of the three individual sets of regions to obtain a combined perceptuo-motor network. In a third step, we compared our meta-analytically derived task-based networks to three RSFC-based data-driven network definitions from the literature. Lastly, we computed the minimum z-statistic of our three meta-analytically derived brain networks to investigate common, domain-unspecific brain regions between these networks.

Our analyses mostly yielded regions that are well known to be recruited for the basic perceptual and motor processes of our interest. To our knowledge, there is no meta-analytically defined visual network published yet. In our analysis, convergence was found in visual cortices as well as frontoparietal brain regions including right ventral PMd/lateral FEF and bilateral IFJ. While ventral PMd/lateral FEF is assumed to be involved in controlling targeted eye movements^30,31^, bilateral IFJ was recently found to be consistently activated during (emotional) face processing^32^, possibly in relation to automatic processes of visual attentional orienting.

When comparing our resulting set of regions related to auditory processing with Petacchi et al.’s^7^ earlier meta-analytic results, which were based on a manual selection from the then available body of pertinent literature and also tested against rest or low-level control conditions, 4 of their 11 result coordinates overlap with our set of regions: their 3 auditory cortex coordinates as well as their coordinate in right aIns. Regions that do not match with our resulting clusters are right middle frontal gyrus and right IPL. Our analysis resulted in different clusters of convergence within the cerebellum and additional regions of convergence such as left aIns, pallidum, putamen, thalamus, SMA, pre-SMA, and PMd.

Our results on motor-related brain activity agree well with Hardwick et al.’s^6^ meta-analysis on brain regions involved in movement execution, which was based on a manually selected sample of all eligible studies available at that time. As compared to our findings, Hardwick et al. reported additional convergence in left operculum and more lateral convergence in right postcentral gyrus extending into parietal opercular cortex. Conversely, our results included additional clusters of convergence in bilateral aINS and right IPL.

A possible reason for these differences between our BrainMap-based meta-analytical results and earlier meta-analyses on manually selected studies might be the number of experiments used for analysis. While Hardwick et al.^6^ included somewhat more than half as many (n = 142) experiments as our analysis did (n = 251), Petacchi et al.^7^ included only 27 experiments, which is less than a quarter of our number of experiments (n = 122). Further, topic-based, manually selected neuroimaging meta-analyses often do not only come at some expense for sample size but also heterogeneity.

Comparing our meta-analytic findings to data-driven RSFC-based network definitions, we found a large degree of overlap in expected areas associated with basic visual, auditory or motor processing. This agreement corroborates the notion that brain networks are rather consistently organized across states of task and “rest”^17–20^. However, as compared to RSFC-derived networks, our task-based results appear to be more specific and included additional, domain-unspecific regions (e.g., IFJ, preSMA, aIns) associated with “higher-order” cognitive functions. This suggests that during tasks, perceptuo-motor networks are recruited alongside supramodal, integrative regions thought to be associated with functions like cognitive control. One possible explanation could be an adaptive functional brain organization that adjusts network topology depending on task demands. For instance, Di et al.^33^ found more between-network than within-network connections during task performance (vs. resting state) as well as a hub shift during task states. Similar to our results and the comparison of our results to RSFC-based network definitions, these authors specifically found the thalamus to have a stronger coactivation profile throughout the brain (i.e., a higher number of coactivations with other brain regions) during task, relative to rest. The authors figured that the thalamus mediated cortico-cortical communication during tasks. Left aIns and preSMA, which are consistently implicated in our perceptuo-motor networks, may be similarly involved in mediating cortico-cortical communication during task states. aIns is supposed to play a pivotal role in monitoring and implementing relevant task sets^34–37^, while preSMA has been linked to cognitive action control and motor preparation^38–40^. These regions are part of what Duncan and his collaborators^41–43^ called “multiple-demand” brain system, which has been defined as brain regions consistently recruited during all kinds of cognitively demanding tasks. Furthermore, task context effects (e.g., how an instruction is presented) have to be considered. Taken together, these findings support the view of brain networks as entities that are not strictly separate but can be (partly) combined, disconnected, and re-combined to generate the neural circuitry necessary to subserve the particular cognitive function at hand.

Very recently, NeuroQuery, a new data-driven approach to meta-analysis, has been proposed^44^. There, in contrast to traditional coordinate-based meta-analytic frameworks, supervised machine learning is applied to predict brain maps given any combination of neuroscience-related terms. A major difference to classic topic-based meta-analyses is that NeuroQuery weighs and combines terms to predict brain locations most likely to be reported in a study, rather than isolating certain terms and evaluating the across-study convergence of activations linked to a given topic (or set of terms). NeuroQuery thus addresses the lack of a universally established vocabulary to describe cognitive processes and functions, offering a data-driven solution. We compared our coordinate-based meta-analytic results to those obtained with this new approach. To this end, we used the same paradigm classes chosen for our BrainMap-based analysis as query terms in NeuroQuery, plus the terms “healthy” and “adult.” The results of both approaches showed large overlap. However, the NeuroQuery approach did not yield any subcortical or cerebellar regions for visual and auditory processing and hardly any (only smaller parts of left thalamus and putamen) for processes related to motor execution. However, despite these minor discrepancies, the overall convergence of both approaches further supports the validity of our BrainMap-derived delineation of functional networks in the human brain.

The goal of this study was to meta-analytically define sets of brain regions that are reliably associated with visual, auditory or motor-related processing. Our results are made publicly available via the ANIMA database^45^ for use in subsequent research. As alluded to above, studying associations between brain networks and behavior might benefit from including regions related to perceptuo-motor processes, as task performance may be influenced by input- or output-related processing^4,46^. Thus, including perceptuo-motor regions to capture the neural correlates of these aspects of task processing may allow drawing a more complete and accurate picture of brain-behavior relationships.

A limitation of the current study was the tradeoff between quantity and quality of the experiments included. In particular, although all contrasts were included based on carefully defined inclusion and exclusion criteria and further manually screened in the Sleuth workspace, our semi-automated sampling from the BrainMap database prevented detailed manual checking of how well each individual contrast reflected the process of interest. This contrasts with typical topic-based neuroimaging meta-analyses, in which stricter eligibility checks should be the rule (see^21^), which, however, often comes at some expense for sample size and heterogeneity. Furthermore, due to the automated extraction of experiments via Sleuth, it was impossible to control for sample overlap; that is, multiple experiments from one study were not pooled to constitute a single experiment^11^. However, given the quantity of studies and experiments (see Fig. 1), it is unlikely that study-specific biases might have had a significant influence on the results. Further, the BrainMap database does not contain all neuroimaging studies available. However, with including approximately 1/4^th^ of eligible neuroimaging studies (i.e., studies reporting peak activation locations in stereotaxic coordinates) across the full range of topics studied, BrainMap allows powerful, large-scale meta-analyses, yielding robust results that generalize well. It was the aim of this study to meta-analytically capture the neural correlates of “basic” perceptuo-motor processes through including a great diversity of tasks and including only contrasts against a resting baseline condition. Having high variability across tasks but low variability in the comparative baseline condition allowed us to distill the neural correlates of the fundamental processes that these tasks share, per domain: “basic” visual, auditory or motor processing. The regions of convergence known to be associated with higher-order cognitive functioning were indeed found to be driven by many and very diverse experiments and were not generally associated with more demanding or complex tasks of our sample.

In summary, based on three comprehensive coordinate-based neuroimaging meta-analyses, we provide a robust quantitative synthesis of the neuroimaging literature on visual, auditory, or motor-related processing in the human brain. These reliable definitions of domain-typical functional brain networks as well as a combined perceptuo-motor network are made publicly available via the ANIMA database (https://anima.inm7.de) for the benefit of future research.

## Supplementary Information

Supplementary Information.

## Data Availability

Imaging data was obtained from the BrainMap (http://www.brainmap.org) database. Sleuth (http://www.brainmap.org/sleuth/) workflows (i.e., experiments that were included for analyses) as well as the maps of the derived brain networks are made publicly available via the ANIMA database (https://anima.inm7.de). The meta-analyses were conducted using the revised version of the ALE algorithm for coordinate-based meta-analysis of neuroimaging results as implemented in GingerALE 3.0.2 (http://www.brainmap.org/ale/).
