# Supplementary material for: Delineating visual, auditory and motor regions in the human brain with functional neuroimaging: a BrainMap-based meta-analytic synthesis
Source: Sci Rep. 2021 May 11;11:9942. doi: 10.1038/s41598-021-88773-9 (PMC8113600; doi:10.1038/s41598-021-88773-9)
Supplement: Supplementary file 1 — Supplementary Information. [file 41598_2021_88773_MOESM1_ESM.pdf]

Delineating visual, auditory and motor regions in the human brain with functional neuroimaging: a BrainMap-based meta-analytic synthesis

Marisa K. Heckner[1,2*], Edna C. Cieslik[1,2], Vincent Küppers[1,2], Peter T. Fox[3], Simon B. Eickhoff[1,2], & Robert Langner[1,2*].

[1]Institute of Neuroscience and Medicine (INM-7: Brain and Behaviour), Research Centre Jülich, Jülich, Germany

[2]Institute of Systems Neuroscience, Medical Faculty, Heinrich Heine University Düsseldorf, Düsseldorf, Germany

[3]Research Imaging Center, University of Texas Health Science Center, San Antonio, TX, United States

*Corresponding authors: Marisa Heckner or Robert Langner, Institute of Neuroscience and Medicine (INM-7), Research Centre Jülich, 52428 Jülich, Germany, tel.: +49 2461 61 6582, e-mail: m.heckner@fz-juelich.de (M.K.H.); r.langner@fz-juelich.de (R.L.)

Supplementary Material

Activation Likelihood Estimation

The meta-analyses were conducted using the revised version of the ALE algorithm for coordinate-based meta-analysis of neuroimaging results as implemented in GingerALE 3.0.2 (1,2,3). This algorithm aims to identify areas with across-experiment activity convergence that is higher than expected from random spatial association. ALE models the activation coordinates included as centers of 3-D Gaussian probability distributions to acknowledge the spatial uncertainty associated with each focus, which is weighted according to the number of participants per experiment. The probability distributions of all activation foci of a given experiment are then combined for each voxel, which creates a modeled activation (MA) map. Taking the union across these MA maps of all experiments included, yields voxel-wise ALE scores describing the convergence of results (i.e., the estimated activation likelihood) across studies at each particular location of the brain. These ALE scores are then compared to an empirical null distribution reflecting random spatial associations between all MA maps to distinguish "true" convergence across studies from random convergence. In particular, the null hypothesis was derived by sampling a voxel at random from each of the MA maps and taking the union of their probability values in the same manner as for the (spatially contingent) voxels in the original analysis. The *p*-value of a "true" ALE score was then given by the proportion of equal or higher values obtained under the null distribution.

Before analysis, any coordinates reported in Talairach space were transformed into MNI space (4,5). The resulting non-parametric *p*-values for each meta-analysis were cut off at a threshold of $p < 0.05$ (cluster inclusion threshold at voxel level: $p < 0.001$). Cluster level family-wise error correction was done by first, thresholding the statistical image of the uncorrected

voxel-wise p-values of the original analyses at cluster-forming threshold $p < 0.001$. And second, comparing the size of the clusters surviving this threshold against a null-distribution of cluster-sizes. The latter was derived by simulating 1,000 datasets of randomly distributed foci but with otherwise identical properties (e.g., number of foci) as the original dataset.

Table S1. Checklist for Neuroimaging Meta-Analyses.

The research question was specifically defined	YES, and it included the following contrasts:<br><br>1) task > baseline/rest
The literature search was systematic	YES, it included the following search criteria in BrainMap:<br><br>Experiment<br>1) Context *is* normal mapping<br>2) Activation *is* activation only<br>3) Imaging modality *is* fMRI or PET<br>4) Control *is* low level<br>5) Contrast *is not* subject group<br>6) Contrast *is not* external variable<br><br>Subject<br>1) Age *is more than* 17<br><br>Paradigm Classes<br>1) Visual: face monitor/discrimination, film viewing, fixation, flashing checkerboard, passive viewing, visual object identification, visual pursuit/tracking, visuospatial attention<br>2) Auditory: divided auditory attention, music comprehension, oddball discrimination, passive listening, phonological discrimination, pitch monitor/discrimination, tone monitor/discrimination<br>3) Motor: writing, chewing/swallowing, drawing, isometric force, motor learning, grasping, finger tapping/button press, flexion/extension
Detailed inclusion and exclusion criteria were applied	YES, and reasons of non-standard criteria were:<br><br>Manual exclusion of:<br>- Active control which leads to subtraction of process of interest<br>- Single subject experiments

	- Conjunction/main effect/pooled effects when single experiments were already included<br>- Equal experiments from same study<br>- Task did not fit peripheral process of interest<br>- Correlation with external variable<br>- Contrast between subject groups<br>- Unclear contrast
Sample overlap was taken into account	NO
All experiments used the same search coverage (state how brain coverage was assessed and how small volume corrections and conjunctions were taken into account)	YES, the search coverage was the following:<br>- Only whole-brain coverage<br>- Exclusion of ROI studies
Studies are converted to a common reference space	YES, using the following conversion(s):<br>- Coordinates reported in Talairach space were converted to MNI space (5)
The study protocol and all analyses was planned beforehand, including the methods and parameters used for inference, correction for multiple testing, etc.	YES:<br>1) No non-planned or post-hoc analyses.<br>2) The meta-analysis used the default methods and parameters of GingerALE and our group.
The paper includes meta-analytic diagnostics	NO

Table S2. Brain Regions Showing Significant Convergence of Activity for Visual Processing.

ALE-Analysis	Voxel	MNI Coordinates			Z_{max}	Cytoarchitectonic Assignment (Overlap in %[a])	Macroanatomical Region (Overlap in %[a])
		x	y	z			
Visual	4437	44	-76	-8	6.44	Area hOc4la (64.2)	Lateral Occipital Cortex, inferior division (66.0)
						Area FG2 (17.9)	Occipital Fusiform Gyrus (5.0)
						Area FG1 (4.0)	
						Area hOc4lp (2.2)	
						Area hOc4v (V4(v); 2.0)	
		18	-86	-10	6.29	Area hOc3v (V3v; 49.6)	Occipital Fusiform Gyrus (42.0)
						Area hOc2 (V2; 31.3)	Occipital Pole (12.0)
						Area hOc4v (V4(v); 4.6)	
		-36	-76	-4	6.17		Lateral Occipital Cortex, inferior division (18.0)
							Occipital Fusiform Gyrus (11.0)
		42	-50	-22	6.10	Area FG4 (96.0)	Temporal Occipital Fusiform Cortex (71.0)
						Area FG2 (3.3)	Inferior Temporal Gyrus, temporooccipital part (7.0)
		-24	-92	-2	6.07	Area hOc4lp (52.1)	Occipital Pole (20.0)
						Area hOc1 (V1; 3.6)	Lateral Occipital Cortex, inferior division (14.0)
		-22	-94	0	NA	Area hOc4lp (44.7)	Occipital Pole (27.0)
						Area hOc1 (V1; 5.1)	Lateral Occipital Cortex, inferior division (8.0)
		-38	-78	-8	NA	Area FG1 (27.3)	Occipital Fusiform Gyrus (23.0)
						Area FG2 (26.0)	Lateral Occipital Cortex, inferior division (22.0)
						Area hOc4la (12.9)	
						Area hOc4v (V4(v); 9.3)	
		-12	-80	-10	5.74	Area hOc3v (V3v; 53.4)	Lingual Gyrus (42.0)
						Area hOc2 (V2; 25.5)	Occipital Fusiform Gyrus (26.0)
						Area hOc1 (V1; 13.0)	
						Area hOc4v (V4(v); 8.1)	
		-40	-72	-12	NA	Area FG2 (48.4)	Occipital Fusiform Gyrus (30.0)
						Area FG1 (25.8)	Lateral Occipital Cortex, inferior division (22.0)
						Area hOc4la (10.7)	
		-40	-84	4	5.37	Area hOc4la (68.0)	Lateral Occipital Cortex, inferior division (52.0)

					Area hOc4lp (29.0)	Lateral Occipital Cortex, superior division (7.0)
1110	-6	2	56	6.30	Area 6mr/preSMA (62.4)	Juxtapositional Lobule
					Area 6mc/SMA (3.5)	
	-2	-8	48	5.75		Juxtapositional Lobule (60.0)
						Cingulate Gyrus, anterior division (28.0)
	6	14	48	5.44	Area 6mr/preSMA (18.1)	Paracingulate Gyrus (55.0)
						Superior Frontal Gyrus (14.0)
	-6	8	40	4.97		Cingulate Gyrus, anterior division (48.0)
						Paracingulate Gyrus (24.0)
790	44	6	26	6.87	Area 44 (14.1)	Precentral Gyrus (45.0)
						Inferior Frontal Gyrus, pars opercularis (16.0)
	42	-4	48	5.74	Area 4a (11.6)	Precentral Gyrus (48.0)
						Middle Frontal Gyrus (9.0)
	52	2	38	4.97	Area 44 (7.5)	Precentral Gyrus (47.0)
						Middle Frontal Gyrus (4.0)
	30	-8	50	4.18	Area 6d3 (34.6)	Precentral Gyrus (35.0)
					Area 6d1 (16.2)	Middle Frontal Gyrus (12.0)
500	28	-56	44	5.58	Area hIP3 (IPS; 45.7)	Superior Parietal Lobule (31.0)
					Area hIP1 (IPS; 15.5)	Lateral Occipital Cortex, superior division (17.0)
	20	-60	54	5.55	Area 7A (SPL; 51.8)	Lateral Occipital Cortex, superior division (28.0)
					Area hIP3 (IPS; 33.2)	Superior Parietal Lobule (9.0)
					Area 7P (SPL; 9.1)	
	32	-54	60	3.76	Area 7A (SPL; 29.5)	Superior Parietal Lobule (45.0)
					Area 7PC (SPL; 16.1)	Lateral Occipital Cortex, superior division (11.0)
					Area hIP3 (IPS; 9.1)	
484	-30	-24	52	7.23	Area 4p (5.8)	Precentral Gyrus (33.0)
					Area 3a (2.1)	Postcentral Gyrus (13.0)
	-40	-34	44	4.08	Area 2 (24.5)	Postcentral Gyrus (32.0)
					Area PFt (IPL; 15.1)	Superior Parietal Lobule (18.0)
					Area 5L (SPL; 4.6)	
					Area 3a (2.8)	
	-42	-16	50	3.75		Precentral Gyrus 40.0)
						Postcentral Gyrus (23.0)

	-36	-16	62	3.62	Area 6d1 (42.8)	Precentral Gyrus (26.0)
					Area 4a (1.1)	Postcentral Gyrus (3.0)
464	-24	-64	46	5.59	Area hIP3 (IPS; 27.0)	Lateral Occipital Cortex, superior division (48.0)
					Area hIP6 (IPS; 17.4)	Superior Parietal Lobule (5.0)
					Area 7A (SPL; 6.3)	
	-36	-46	52	3.81	Area 7PC (SPL; 34.6)	Superior Parietal Lobule (43.0)
					Area hIP3 (IPS; 15.7)	Supramarginal Gyrus, posterior division (7.0)
					Area 2 (7.7)	
					Area 5L (SPL; 1.6)	
	-32	-52	54	3.70	Area hIP3 (IPS; 37.9)	Superior Parietal Lobule (47.0)
					Area 7PC (SPL; 32.1)	Lateral Occipital Cortex, superior division (5.0)
					Area 7A (SPL; 25.7)	
	-26	-52	52	3.54	Area hIP3 (IPS; 4.0)	Superior Parietal Lobule (37.0)
					Area 7PC (SPL; 2.9)	Lateral Occipital Cortex, superior division (4.0)
	-10	-66	46	3.41	Area 7A (SPL; 19.9)	Precuneus (31.0)
					Area 7P (SPL; 14.5)	
353	-52	2	30	5.81	Area 44 (11.3)	Precentral Gyrus (51.0)
						Inferior Frontal Gyrus, pars opercularis (4.0)
	-44	4	30	5.61	Area 44 (29.8)	Precentral Gyrus (47.0)
						Middle Frontal Gyrus (13.0)
183	38	28	0	5.13	Area OP9 (69.2)	Frontal Orbital Cortex (37.0)
					Area 45 (11.7)	Frontal Opercular Cortex (19.0)
					Area Id7 (7.7)	
					Area OP8 (3.3)	
	50	30	4	3.44	Area 45 (43.4)	Inferior Frontal Gyrus, pars triangularis (46.0)
					Area OP8 (1.6)	Frontal Pole (8.0)

Note. ALE = activation likelihood estimation; MNI = Montreal Neurological Institute; NA = not applicable; Z_{max} = z-score of the local maximum.

[a]Overlap in % of the resulting coordinate and the assigned cytoarchitectonic/macroanatomical region.

Table S3. Brain Regions Showing Significant Convergence of Activity for Auditory Processing.

ALE-Analysis	Voxel	MNI Coordinates x	y	z	Z_{max}	Cytoarchitectonic Assignment (Overlap in %[a])	Macroanatomical Region (Overlap in %[a])
Auditory	3723	-56	-22	6	14.46	Area TE 1.0 (5.1)	Planum Temporale (50.0)
						Area OP1 (SII; 4.5)	Heschl's Gyrus (15.0)
		-42	-32	12		Area TE 1.1 (65.1)	Planum Temporale (61.0)
						Area OP1 (SII; 19.5)	Parietal Opercular Cortex (12.0)
						Area TE 1.0 (6.5)	
		-32	22	2	6.68	Area Id7 (94.0)	Insular Cortex (67.0)
							Frontal Orbital Cortex (3.0)
		-56	0	-6	5.67	Area TE 3 (5.4)	Superior Temporal Gyrus, anterior division (37.0)
						Area TE 1.2 (4.7)	Planum Temporale (17.0)
		-54	8	4	5.09	Area 44 (36.1)	Precentral Gyrus (41.0)
							Inferior Frontal Gyrus, pars opercularis (35.0)
	3716	56	-22	6	12.21		Planum Temporale (32.0)
							Heschl's Gyrus (5.0)
		36	22	4	6.92	Area OP9 (27.8)	Frontal Opercular Cortex (43.0)
						Area Id7 (26.8)	Insular Cortex (29.0)
						Area 45 (12.9)	
						Area OP8 (8.8)	
		58	-2	-8	6.26	Area TE 3 (46.5)	Superior Temporal Gyrus, anterior division (60.0)
							Superior Temporal Gyrus, posterior division (5.0)
		46	12	4	5.71	Area 44 (20.9)	Frontal Opercular Cortex (41.0)
						Area OP8 (20.3)	Inferior Frontal Gyrus, pars opercularis (15.0)
		56	12	4	5.44	Area 44 (53.0)	Inferior Frontal Gyrus, pars opercularis (54.0)
							Precentral Gyrus (18.0)
		46	-16	-10	5.19	Area Id1 (2.5)	Superior Temporal Gyrus, posterior division (24.0)
							Middle Temporal Gyrus, posterior division (5.0)
	833	-4	0	64	6.96	Area 6mr/preSMA (49.6)	Juxtapositional Lobule (87.0)
						Area 6mc/SMA (15.6)	
		0	10	54	6.51		Paracingulate Gyrus (22.0)

						Superior Frontal Gyrus (12.0)
594	-20	8	4	6.99		Left Putamen (70.2)
	-12	-2	10	5.04		Left Thalamus (42.3)
						Left Caudate (9.9)
	-10	-16	2	4.43		Left Thalamus (99.9)
	-14	-12	14	4.22		Left Thalamus (95.9)
418	22	4	6	6.96		Right Putamen (96.1)
	14	-4	10	4.95		Right Thalamus (49.6)
332	-50	-6	50	5.95		Precentral Gyrus (75.0)
						Middle Frontal Gyrus (2.0)
	-48	-8	40	5.53		Precentral Gyrus (43.0)
300	-30	-66	-26	7.11		Cerebellum Left VI (79.0)
						Cerebellum Left Crus I (20.0)
192	54	0	48	6.56	Area 3b (2.6)	Precentral Gyrus (61.0)
						Middle Frontal Gyrus (3.0)
183	28	-62	-26	5.72		Cerebellum Right VI (88.0)
						Cerebellum Right Crus I (4.0)

Note. ALE = activation likelihood estimation; MNI = Montreal Neurological Institute; Z_{max} = z-score of the local maximum.
[a]Overlap in % of the resulting coordinate and the assigned cytoarchitectonic/macroanatomical region.

Table S4. Brain Regions Showing Significant Convergence of Activity for Motor-Related Processing.

ALE-Analysis	Voxel	MNI Coordinates			Z_{max}	Cytoarchitectonic Assignment (Overlap in %[a])	Macroanatomical Region (Overlap in %[a])
		x	y	z			
Motor	8719	-4	-2	56	17.42	Area 6mr / preSMA (35.7)	Juxtapositional Lobule (85.0)
						Area 6mc / SMA (26.1)	
		-36	-18	58	14.70	Area 6d1 (17.8)	Precentral Gyrus (33.0)
						Area 4a (7.9)	Postcentral Gyrus (2.0)
		-26	-4	62	9.25	Area 6d2 (39.6)	Superior Frontal Gyrus (37.0)
						Area 6d3 (24.2)	Precentral Gyrus (15.0)
						Area 6d1 (12.7)	
		-18	-56	66	8.22	Area 7A (SPL; 51.6)	Superior Parietal Lobule (43.0)
						Area 5L (SPL; 15.1)	Lateral Occipital Cortex, superior division (17.0)
						Area 7PC (SPL; 12.3)	
		-56	4	28	7.86	Area 44 (16.9)	Precentral Gyrus (62.0)
							Inferior Frontal Gyrus, pars opercularis (6.0)
		-52	-8	46	6.97		Precentral Gyrus (68.0)
							Postcentral Gyrus (7.0)
		-48	-28	20	6.88	Area OP1 (SII; 77.4)	Parietal Opercular Cortex (52.0)
						Area OP2 (PIVC; 13.8)	Central Opercular Cortex (12.0)
						Area TE 1.1 (4.2)	
						Area TE 1.0 (3.3)	
		-52	-8	32	6.85	Area 4p (17.7)	Precentral Gyrus (47.0)
						Area 4a (3.7)	Postcentral Gyrus (20.0)
		-56	-18	42	6.04	Area 3b (47.2)	Postcentral Gyrus (69.0)
						Area 1 (38.1)	Supramarginal Gyrus, anterior division (5.0)
						Area 2 (14.8)	
		-36	-40	46	NA	Area hIP3 (IPS; 13.1)	Superior Parietal Lobule (27.0)
						Area 2 (10.3)	Postcentral Gyrus (22.0)
						Area 5L (SPL; 7.6)	
						Area 3a (6.2)	
	5336	26	0	2	9.79		Right Putamen (99.7)

	14	-18	4	9.37		Right Thalamus (99.1)
	26	-4	56	9.31	Area 6d3 (67.6)	Superior Frontal Gyrus (24.0)
					Area 6d1 (8.4)	Middle Frontal Gyrus (19.0)
					Area 6d2 (6.6)	
	38	-16	58	8.68	Area 4a (7.3)	Precentral Gyrus (36.0)
					Area 3b (1.7)	Postcentral Gyrus (3.0)
	42	-42	54	8.42	Area hIP2 (IPS; 36.3)	Superior Parietal Lobule (33.0)
					Area hIP3 (IPS; 24.1)	Supramarginal Gyrus, posterior division (24.0)
					Area 7PC (SPL; 17.3)	
					Area PFm (IPL; 14.0)	
					Area PGa (IPL; 8.2)	
	54	-6	32	7.64	Area 4p (21.7)	Precentral Gyrus (31.0)
					Area 3b (20.6)	Postcentral Gyrus (31.0)
					Area 4a (2.4)	
					Area 1 (1.5)	
	52	-8	36	NA	Area 3b (23.7)	Precentral Gyrus (44.0)
					Area 4p (17.8)	Postcentral Gyrus (28.0)
					Area 4a (10.5)	
	34	22	4	6.68	Area Id7 (42.2)	Insular Cortex (53.0)
					Area OP9 (12.3)	Frontal Opercular Cortex (26.0)
					Area 45 (9.4)	
	60	6	22	6.19	Area 44 (11.5)	Precentral Gyrus (63.0)
						Inferior Frontal Gyrus, pars opercularis (2.0)
	40	-34	48	5.68	Area 2 (67.1)	Postcentral Gyrus (26.0)
					Area 7PC (SPL; 13.2)	Supramarginal Gyrus, anterior division (18.0)
					Area 3b (10.9)	
					Area hIP2 (IPS; 6.5)	
					Area PFt (IPL; 2.0)	
3373	20	-56	-22	13.18		Cerebellum Right VI (86.0)
						Cerebellum Right V (11.0)
	-32	-50	-32	8.22		Cerebellum Left VI (94.0)
						Cerebellum Left Crus I (2.0)
	-16	-60	-22	8.20		Cerebellum Left VI (89.0)

						Cerebellum Left V (7.0)
	-34	-58	-28	7.41		Cerebellum Left VI (78.0)
						Cerebellum Left Crus I (21.0)
	-24	-54	-24	7.37		Cerebellum Left VI (93.0)
						Cerebellum Left V (5.0)
	16	-74	-36	6.16		Cerebellum Right Crus II (73.0)
						Cerebellum Right Crus I (5.0)
	-14	-60	-10	5.15	Area hOc3v (V3v; 13.2)	Lingual Gyrus (39.0)
					Area hOc2 (V2; 2.5)	Cerebellum Left V (14.0)
	-4	-50	-10	4.11		Cerebellum Left I-IV (97.0)
	14	-68	-48	3.98		Cerebellum Right VIIIa (65.0)
						Cerebellum Right VIIb (28.0)
2085	-14	-18	4	12.07		Left Thalamus (99.9)
	-24	-4	2	9.61		Left Putamen (53.9)
						Left Pallidum (46.1)
	-36	18	2	6.21	Area Id7 (41.8)	Insular Cortex (62.0)
					Area OP8 (7.6)	Frontal Opercular Cortex (16.0)
					Area 44 (4.6)	

Note. ALE = activation likelihood estimation; MNI = Montreal Neurological Institute; NA = not applicable; Z_{max} = z-score of the local maximum.

[a]Overlap in % of the resulting coordinate and the assigned cytoarchitectonic/macroanatomical region.

Table S5. Brain Regions Showing Significant Convergence of Activity for the Maximum Z-Statistic of Visual, Auditory, and Motor-Related Processing.

ALE-Analysis	Voxel	MNI Coordinates			Cytoarchitecture (Overlap in %[a])	Macroanatomy (Overlap in %[a])
		x	y	z		
Visual+Auditory+Motor	14885	-4	-2	56	Area 6mr / preSMA (35.7)	Juxtapositional Lobule (85.0)
					Area 6mc / SMA (26.1)	
		-36	-18	58	Area 6d1 (17.8)	Precentral Gyrus (33.0)
					Area 4a (7.9)	Postcentral Gyrus (2.0)
		-56	-22	6	Area TE 1.0 (5.1)	Planum Temporale (50.0)
					Area OP1 (SII; 4.5)	Heschl's Gyrus (15.0)
		-14	-18	4		Left Thalamus (99.9)
		-24	-4	2		Left Putamen (53.9)
						Left Pallidum (46.1)
		-26	-4	62	Area 6d2 (39.6)	Superior Frontal Gyrus (37.0)
					Area 6d3 (24.2)	Precentral Gyrus (15.0)
					Area 6d1 (12.7)	
		-18	-56	66	Area 7A (SPL; 51.6)	Superior Parietal Lobule (43.0)
					Area 5L (SPL; 15.1)	Lateral Occipital Cortex, superior division (17.0)
					Area 7PC (SPL; 12.3)	
		-56	4	28	Area 44 (16.9)	Precentral Gyrus (62.0)
						Inferior Frontal Gyrus, pars opercularis (6.0)
		-42	-32	12	Area TE 1.1 (65.1)	Planum Temporale (61.0)
					Area OP1 (SII; 19.5)	Parietal Opercular Cortex (12.0)
					Area TE 1.0 (6.5)	
		-52	-8	46		Precentral Gyrus (68.0)
						Postcentral Gyrus (7.0)
	9670	56	-22	6		Planum Temporale (32.0)
						Heschl's Gyrus (5.0)
		26	0	2		Right Putamen (99.7)
		14	-18	4		Right Thalamus (99.1)

	26	-4	56	Area 6d3 (67.6)	Superior Frontal Gyrus (24.0)
				Area 6d1 (8.4)	Middle Frontal Gyrus (19.0)
				Area 6d2 (6.6)	
	38	-16	58	Area 4a (7.3)	Precentral Gyrus (36.0)
				Area 3b (1.7)	Precentral Gyrus (3.0)
	42	-42	54	Area hIP2 (IPS; 36.3)	Superior Parietal Lobule (33.0)
				Area hIP3 (IPS; 24.1)	Supramarginal Gyrus, posterior division (24.0)
				Area 7PC (SPL; 17.3)	
				Area PFm (IPL; 14.0)	
				Area PGa (IPL; 8.2)	
	54	-6	32	Area 4p (21.7)	Precentral Gyrus (31.0)
				Area 3b (20.6)	Postcentral Gyrus (31.0)
				Area 4a (2.4)	
				Area 1 (1.5)	
	52	-8	36	Area 3b (23.7)	Precentral Gyrus (44.0)
				Area 4p (17.8)	Postcentral Gyrus (28.0)
				Area 4a (10.5)	
	34	22	4	Area Id7 (42.2)	Insular Cortex (53.0)
				Area OP9 (12.3)	Frontal Opercular Cortex (26.0)
				Area 45 (9.4)	
	60	6	22	Area 44 (11.5)	Precentral Gyrus (63.0)
					Inferior Frontal Gyrus, pars opercularis (2.0)
7870	20	-56	-22		Cerebellum Right VI (86.0)
					Cerebellum Right V (11.0)
	-32	-50	-32		Cerebellum Left VI (94.0)
					Cerebellum Left Crus I (2.0)
	-16	-60	-22		Cerebellum Left VI (89.0)
					Cerebellum Left V (7.0)
	-34	-58	-28		Cerebellum Left VI (78.0)
					Cerebellum Left Crus I (21.0)
	-24	-54	-24		Cerebellum Left VI (93.0)
					Cerebellum Left V (5.0)
	16	-74	-36		Cerebellum Right Crus II (73.0)

				Cerebellum Right Crus I (5.0)
-30	-66	-26		Cerebellum Left VI (79.0)
				Cerebellum Left Crus I (20.0)
-14	-60	-10	Area hOc3v (V3v; 13.2)	Lingual Gyrus (39.0)
			Area hOc2 (V2; 2.5)	Cerebellum Left V (14.0)
44	-76	-8	Area hOc4lp (64.2)	Lateral Occipital Cortex, inferior division (66.0)
			Area FG2 (17.9)	Occipital Fusiform Gyrus (5.0)
			Area FG1 (4.0)	
			Area hOc4lp (2.2)	
			Area hOc4v (V4(v); 2.0)	
18	-86	-10	Area hOc3v (V3v; 49.6)	Occipital Fusiform Gyrus (42.0)
			Area hOc2 (V2; 31.3)	Occipital Pole (12.0)
			Area hOc4v (V4(v); 4.6)	

Note. ALE = activation likelihood estimation; MNI = Montreal Neurological Institute.
[a]Overlap in % of the resulting coordinate and the assigned cytoarchitectonic/macroanatomical region.

Table S6. Brain Regions Showing Significant Convergence of Activity for the Minimum Z-Statistic of Visual, Auditory, and Motor-Related Processing.

ALE-Analysis	Voxel	MNI Coordinates			Cytoarchitecture (Overlap in %[a])	Macroanatomy (Overlap in %[a])
		x	y	z		
Visual⌒Auditory⌒Motor	406	-2	6	54		Juxtapositional Lobule (53.0)
						Paracingulate Gyrus (14.0)
		-4	2	58	Area 6mr/preSMA (59.9)	Juxtapositional Lobule (81.0)
					Area 6mc/SMA (6.7)	Superior Frontal Gyrus (2.0)
	84	52	2	42	Area 44 (11.8)	Precentral Gyrus (55.0)
					Area 4a (3.3)	Middle Frontal Gyrus (7.0)
		60	6	22	Area 44 (11.5)	Precentral Gyrus (63.0)
	61	38	26	2	Area OP9 (55.0)	Frontal Opercular Cortex (37.0)
					Area 45 (13.6)	Frontal Orbital Cortex (22.0)
					Area OP8 (11.6)	
					Area Id7 (5.4)	
	1	-48	-2	36		Precentral Gyrus (60.0)
						Middle Frontal Gyrus (3.0)

Note. ALE = activation likelihood estimation; MNI = Montreal Neurological Institute.
[a]Overlap in % of the resulting coordinate and the assigned cytoarchitectonic/macroanatomical region.

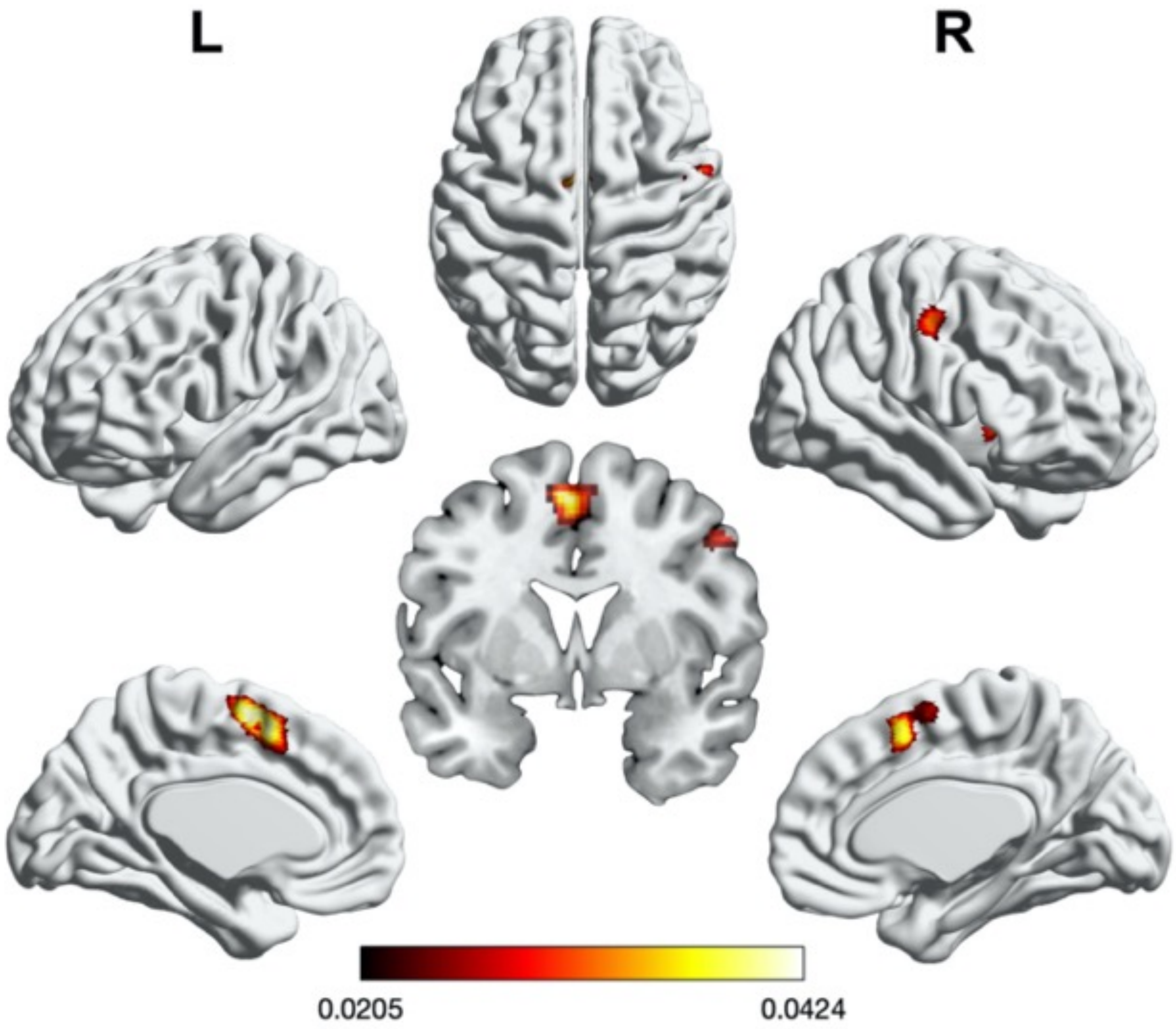

Figure S1. Minimum z-statistic conjunction of the results of three meta-analyses on visual, auditory, and motor-related processing. The scale bar reflects the maximum statistic of the activation likelihood estimation scores. Cortical and cerebellar clusters of convergence were visualized with the BrainNet Viewer (6); subcortical clusters were rendered on the individual anatomical template (“ch2better”) provided with MRIcron (7).

References

1. Eickhoff, S. B. *et al.* Coordinate-based activation likelihood estimation meta-analysis of neuroimaging data: A random-effects approach based on empirical estimates of spatial uncertainty. *Hum. Brain Mapp.* **30,** 2907–2926 (2009).
2. Eickhoff, S. B., Bzdok, D., Laird, A. R., Kurth, F. & Fox, P. T. Activation likelihood estimation meta-analysis revisited. *NeuroImage* **59,** 2349–2361 (2012).
3. Turkeltaub, P. E. *et al.* Minimizing within-experiment and within-group effects in activation likelihood estimation meta-analyses. *Hum. Brain Mapp.* **33,** 1–13 (2012).
4. Laird, A. R. *et al.* Comparison of the disparity between Talairach and MNI coordinates in functional neuroimaging data: validation of the Lancaster transform. *NeuroImage* **51,** 677–683 (2010).
5. Lancaster, J. L. *et al.* Bias between MNI and Talairach coordinates analyzed using the ICBM-152 brain template. *Hum. Brain Mapp.* **28,** 1194–1205 (2007).
6. Xia, M., Wang, J. & He, Y. BrainNet Viewer: a network visualization tool for human brain connectomics. *PloS One* **8,** e68910–e68910 (2013).
7. Rorden, C. & Brett, M. Stereotaxic Display of Brain Lesions. *Behav. Neurol.* **12,** 421719 (1900).
